# Supplementary figures and images for: Bioinformatical analysis identifies PDLIM3 as a potential biomarker associated with immune infiltration in patients with endometriosis
Source: PeerJ. 2022 Mar 30;10:e13218. doi: 10.7717/peerj.13218 (PMC8976475; doi:10.7717/peerj.13218)

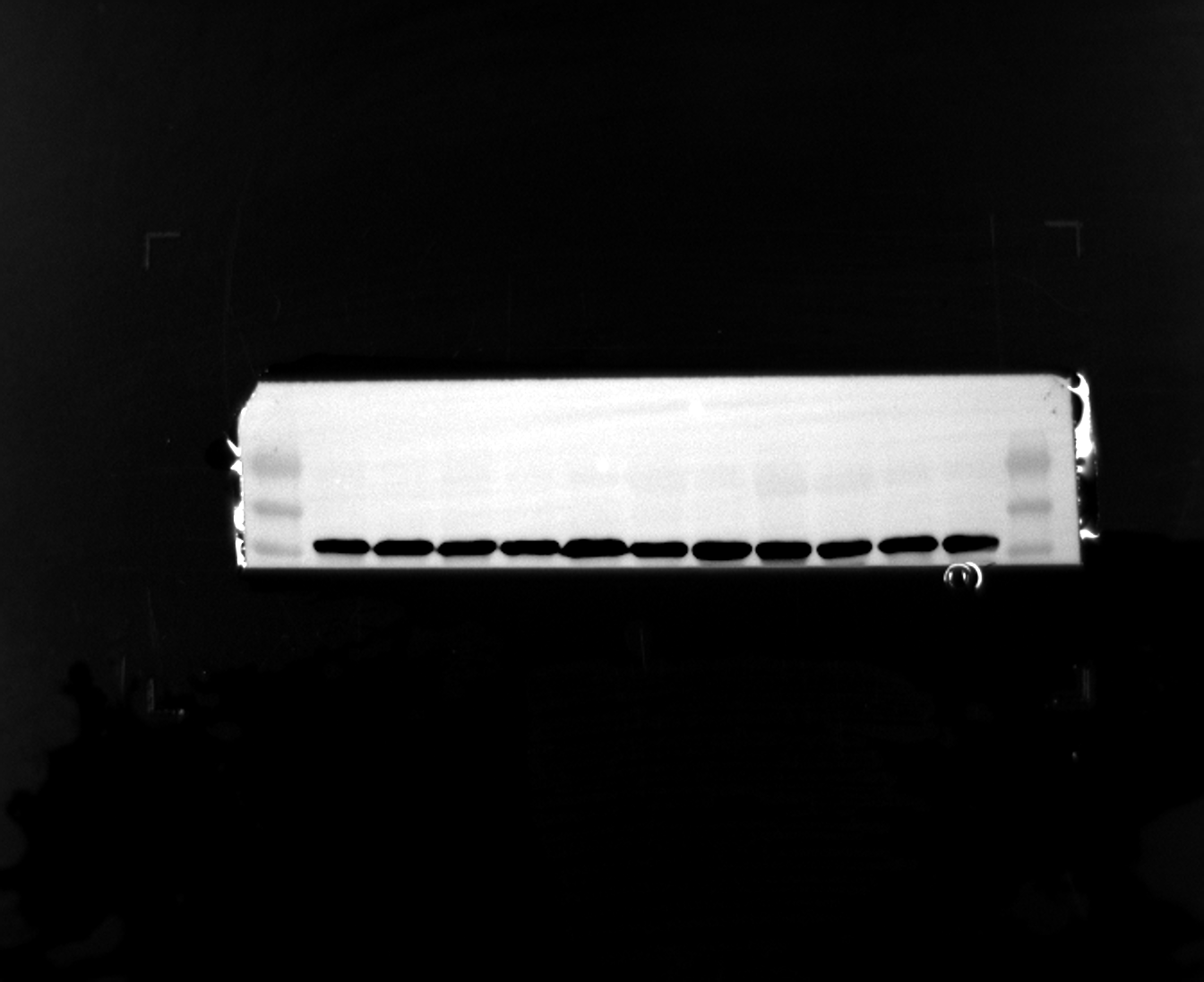

Supplement: Supplemental Information 1 — note: the gene "PINR-LEI" is the target gene "PDLIM3" [file peerj-10-13218-s001.zip › raw data/wb/1-ACTIN.Tif]

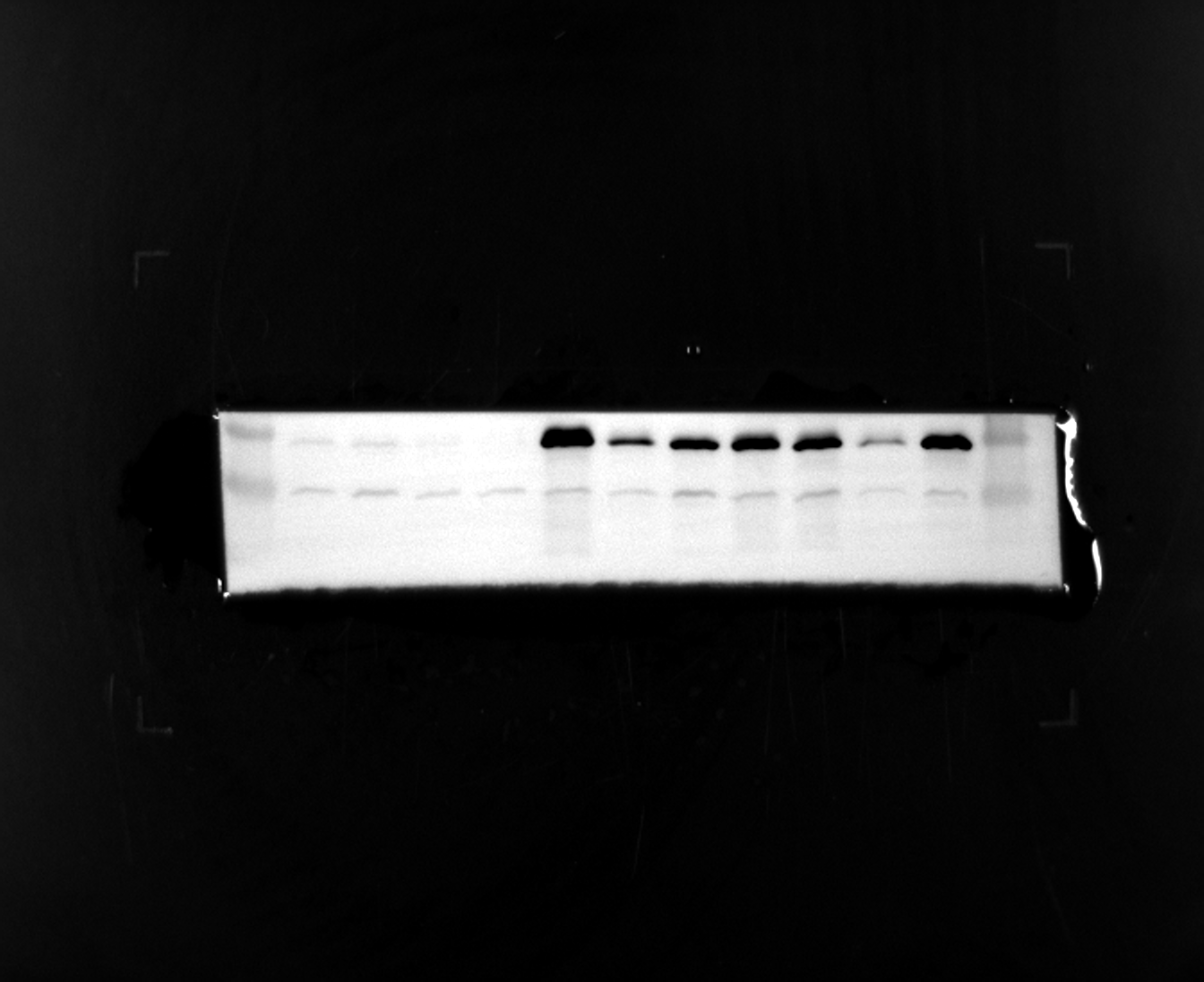

Supplement: Supplemental Information 1 — note: the gene "PINR-LEI" is the target gene "PDLIM3" [file peerj-10-13218-s001.zip › raw data/wb/1-ALP.Tif]

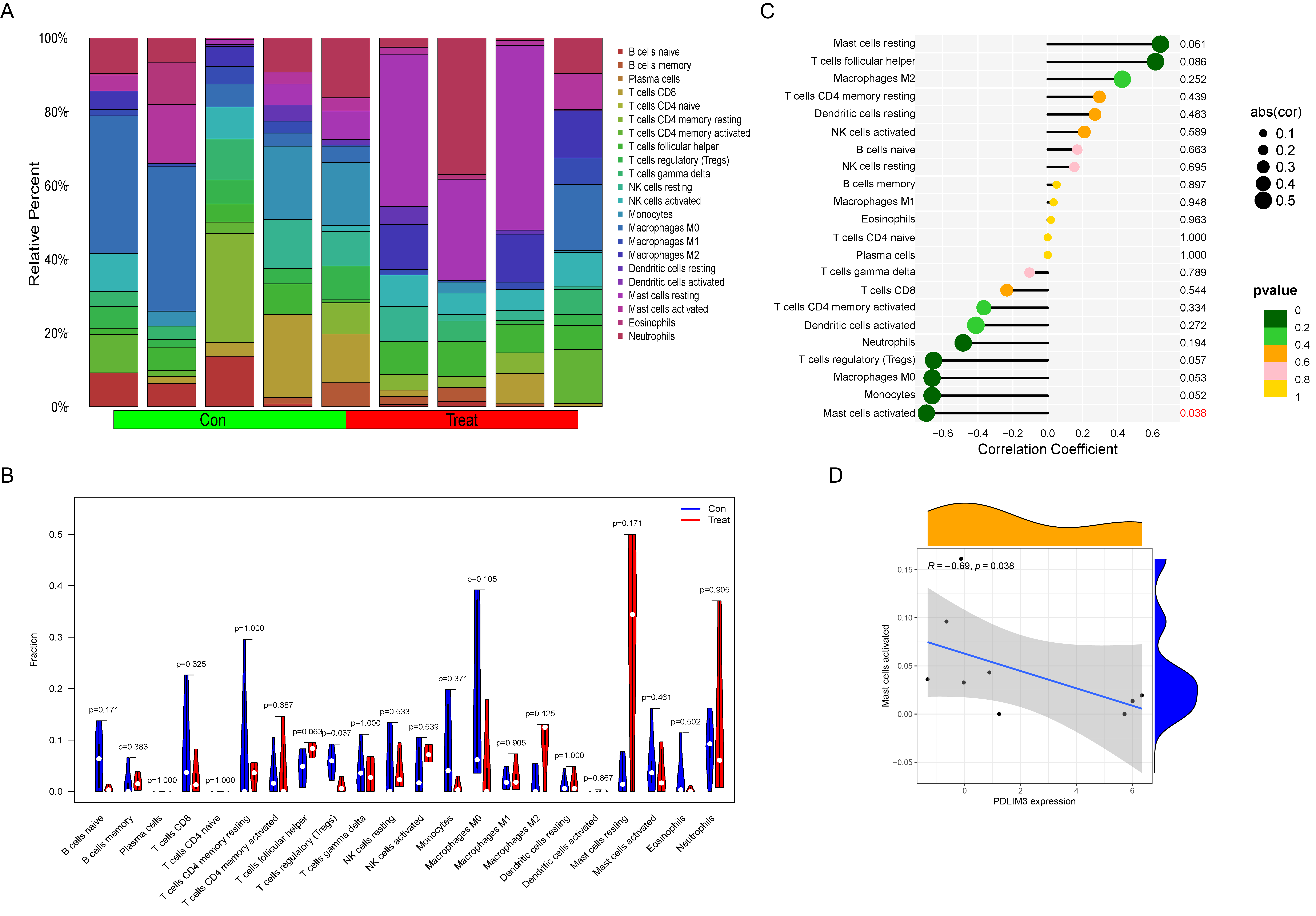

Supplement: Supplemental Information 2 — (A) The composition of immune cells in endometriosis tissues and normal control tissues. (B) Comparison of 22 immune cell subtypes between endometriosis tissues and normal tissues. Blue and red colors represent normal and endometriosis samples, respectively. (C) A comprehensive correlation analysis of PDLIM3 and infiltrating immune cells in endometriosis. (D) Correlation between PDLIM3 and activated mast cell. [file peerj-10-13218-s002.png]
