# Supplementary material for: Bioinformatical analysis identifies PDLIM3 as a potential biomarker associated with immune infiltration in patients with endometriosis
Source: PeerJ. 2022 Mar 30;10:e13218. doi: 10.7717/peerj.13218 (PMC8976475; doi:10.7717/peerj.13218)
Supplement: Supplemental Information 3 [file peerj-10-13218-s003.docx]

Supplementary Table 1. The statistical metrics for key differentially expressed genes (DEGs).

| **Gene Symbol** | **logFC** | **P.Value** | **adj.P.Val** | **t value** | **Regulation** |
| --- | --- | --- | --- | --- | --- |
| CYP17A1 | 8.436731 | 5.50E-08 | 0.00124 | 1.35E+01 | up |
| INSL3 | 7.824079 | 1.99E-07 | 0.00124 | 1.19E+01 | up |
| FLJ38894 | 7.027081 | 4.37E-07 | 0.00124 | 1.09E+01 | up |
| GATA4 | 6.915814 | 3.69E-07 | 0.00124 | 1.11E+01 | up |
| KLHDC8A | 6.879441 | 2.76E-07 | 0.00124 | 1.15E+01 | up |
| PNOC | 6.793546 | 3.67E-07 | 0.00124 | 1.11E+01 | up |
| C7 | 6.781819 | 4.61E-07 | 0.00124 | 1.09E+01 | up |
| NR5A1 | 6.693323 | 1.84E-06 | 0.00231 | 9.42 | up |
| DHRS2 | 6.630581 | 4.22E-07 | 0.00124 | 1.10E+01 | up |
| DLK1 | 6.61175 | 1.42E-06 | 0.00219 | 9.68 | up |
| SPRR2F | 6.428054 | 6.38E-07 | 0.00152 | 1.05E+01 | up |
| TCEAL2 | 6.236489 | 1.10E-06 | 0.00198 | 9.95 | up |
| WISP2 | 6.158233 | 4.20E-06 | 0.00317 | 8.64 | up |
| IGKV1-5 | 6.079094 | 1.69E-06 | 0.0023 | 9.51 | up |
| PRELP | 5.937884 | 1.17E-06 | 0.00198 | 9.88 | up |
| IGKC | 5.909954 | 1.17E-06 | 0.00198 | 9.89 | up |
| MCHR1 | 5.835405 | 1.58E-06 | 0.0023 | 9.58 | up |
| LOC387758 | 5.77045 | 3.90E-06 | 0.00305 | 8.7 | up |
| IGLV2-14 | 5.668364 | 2.25E-06 | 0.00247 | 9.23 | up |
| CLDN11 | 5.667185 | 2.20E-06 | 0.00247 | 9.25 | up |
| VIT | 5.624707 | 3.01E-06 | 0.00279 | 8.95 | up |
| TCF21 | 5.612971 | 2.02E-05 | 0.00644 | 7.27 | up |
| STAR | 5.539658 | 2.26E-06 | 0.00247 | 9.22 | up |
| SPRR2A | 5.495751 | 8.60E-06 | 0.00403 | 7.99 | up |
| GPC3 | 5.463708 | 6.63E-06 | 0.00375 | 8.22 | up |
| CD22 | 5.402438 | 3.73E-06 | 0.00305 | 8.75 | up |
| MYH11 | 5.381513 | 7.10E-06 | 0.00375 | 8.16 | up |
| IGLC2 | 5.357879 | 4.45E-06 | 0.00321 | 8.58 | up |
| GSTA1 | 5.343817 | 1.00E-05 | 0.00438 | 7.86 | up |
| HSD3B2 | 5.323811 | 4.41E-06 | 0.00321 | 8.59 | up |
| PLA2G5 | 5.29563 | 4.26E-06 | 0.00317 | 8.62 | up |
| LOC652848 | 5.288996 | 8.75E-06 | 0.00403 | 7.98 | up |
| CPXM2 | 5.220783 | 4.25E-06 | 0.00317 | 8.62 | up |
| LOC129293 | 5.175516 | 5.93E-06 | 0.00366 | 8.32 | up |
| AMHR2 | 5.154293 | 3.16E-05 | 0.00777 | 6.91 | up |
| C4BPB | 5.146167 | 7.42E-06 | 0.0038 | 8.12 | up |
| FAM70A | 5.129263 | 1.51E-05 | 0.00536 | 7.51 | up |
| CHIT1 | 5.082532 | 6.52E-06 | 0.00375 | 8.24 | up |
| GSTA2 | 5.059461 | 6.07E-06 | 0.00366 | 8.3 | up |
| SIGLEC11 | 4.977402 | 1.49E-05 | 0.00536 | 7.52 | up |
| ACTG2 | 4.972618 | 5.78E-06 | 0.00366 | 8.34 | up |
| PDE1B | 4.936143 | 1.27E-05 | 0.00517 | 7.66 | up |
| RHBG | 4.926517 | 2.65E-05 | 0.0073 | 7.05 | up |
| IGLC1 | 4.91862 | 1.43E-05 | 0.00536 | 7.55 | up |
| ENPP6 | 4.777077 | 3.54E-05 | 0.00826 | 6.83 | up |
| NR1H4 | 4.764608 | 8.60E-05 | 0.01286 | 6.15 | up |
| NFASC | 4.739082 | 1.54E-05 | 0.00536 | 7.5 | up |
| SCN7A | 4.707943 | 1.85E-04 | 0.01962 | 5.61 | up |
| PDLIM3 | 4.655097 | 2.35E-05 | 0.00699 | 7.15 | up |
| DES | 4.647236 | 3.92E-05 | 0.00885 | 6.75 | up |
| PDZRN4 | 4.641851 | 2.94E-05 | 0.0074 | 6.97 | up |
| CAMK1G | 4.592664 | 2.58E-05 | 0.00724 | 7.08 | up |
| ANGPTL5 | 4.481915 | 1.71E-04 | 0.01873 | 5.67 | up |
| VCAM1 | 4.474139 | 1.51E-04 | 0.01742 | 5.75 | up |
| C8orf49 | 4.45048 | 2.08E-05 | 0.00662 | 7.25 | up |
| CCDC3 | 4.444907 | 1.69E-05 | 0.00566 | 7.42 | up |
| MGC10981 | 4.428294 | 6.05E-05 | 0.01064 | 6.41 | up |
| PLP1 | 4.397314 | 5.28E-05 | 0.00999 | 6.52 | up |
| LOC647134 | 4.391773 | 1.98E-05 | 0.00642 | 7.29 | up |
| FMO2 | 4.376385 | 2.79E-05 | 0.00738 | 7.01 | up |
| IGLJ3 | 4.373006 | 1.17E-04 | 0.01528 | 5.93 | up |
| NGFB | 4.364117 | 1.96E-05 | 0.00639 | 7.3 | up |
| RGAG1 | 4.363084 | 5.63E-05 | 0.01008 | 6.47 | up |
| CSTA | 4.358704 | 1.73E-04 | 0.01892 | 5.66 | up |
| MMP23B | 4.356707 | 5.58E-05 | 0.01008 | 6.48 | up |
| CHL1 | 4.338172 | 4.93E-05 | 0.00986 | 6.57 | up |
| ITIH3 | 4.332574 | 2.20E-05 | 0.00684 | 7.2 | up |
| ITGA11 | 4.310458 | 2.58E-05 | 0.00724 | 7.08 | up |
| COLEC11 | 4.302789 | 4.15E-05 | 0.00911 | 6.7 | up |
| ADH1B | 4.285414 | 5.21E-05 | 0.00999 | 6.53 | up |
| CYP11A1 | 4.284405 | 2.40E-05 | 0.00707 | 7.13 | up |
| C18orf34 | 4.284392 | 8.89E-05 | 0.01307 | 6.13 | up |
| WFIKKN2 | 4.279901 | 2.43E-05 | 0.00707 | 7.12 | up |
| HAS1 | 4.275645 | 4.81E-05 | 0.0098 | 6.59 | up |
| MKX | 4.237253 | 4.25E-04 | 0.03153 | 5.05 | up |
| ITLN1 | 4.222441 | 3.98E-05 | 0.00889 | 6.73 | up |
| FABP4 | 4.217983 | 8.14E-05 | 0.01244 | 6.2 | up |
| ARX | 4.195395 | 2.33E-04 | 0.02212 | 5.45 | up |
| GATA6 | 4.194661 | 1.34E-04 | 0.01622 | 5.84 | up |
| COL10A1 | 4.185344 | 1.21E-04 | 0.01565 | 5.91 | up |
| CDON | 4.171995 | 1.17E-04 | 0.01528 | 5.93 | up |
| SFRP2 | 4.169355 | 3.46E-05 | 0.00811 | 6.84 | up |
| TDRD10 | 4.168072 | 2.87E-05 | 0.00739 | 6.99 | up |
| CCDC48 | 4.134485 | 4.64E-05 | 0.00966 | 6.62 | up |
| IGHM | 4.130298 | 3.24E-05 | 0.00785 | 6.89 | up |
| CCL18 | 4.125615 | 3.83E-05 | 0.00881 | 6.76 | up |
| CTSG | 4.094392 | 4.21E-05 | 0.0092 | 6.69 | up |
| ADH1A | 4.065391 | 5.41E-05 | 0.01004 | 6.5 | up |
| IGHG1 | 4.057343 | 4.53E-05 | 0.00954 | 6.63 | up |
| FLJ90650 | 4.044237 | 1.32E-04 | 0.01615 | 5.85 | up |
| CDH3 | 4.014949 | 8.62E-05 | 0.01286 | 6.15 | up |
| APOA1 | 3.992248 | 4.92E-05 | 0.00986 | 6.57 | up |
| LOC651928 | 3.991595 | 4.58E-05 | 0.0096 | 6.63 | up |
| OSAP | 3.985631 | 2.70E-04 | 0.02426 | 5.35 | up |
| PTGIS | 3.97454 | 8.07E-05 | 0.01244 | 6.2 | up |
| PROK1 | 3.970185 | 4.86E-05 | 0.0098 | 6.58 | up |
| SNCG | 3.968667 | 9.00E-05 | 0.0131 | 6.12 | up |
| C10orf116 | 3.952139 | 5.14E-05 | 0.00998 | 6.54 | up |
| CRHBP | 3.951865 | 3.30E-04 | 0.02716 | 5.22 | up |
| DUSP26 | 3.951847 | 6.21E-05 | 0.0108 | 6.39 | up |
| FLJ30901 | 3.935764 | 5.98E-05 | 0.01059 | 6.42 | up |
| NR4A3 | 3.935707 | 1.34E-04 | 0.01622 | 5.84 | up |
| KCNA5 | 3.926551 | 1.25E-04 | 0.01584 | 5.88 | up |
| ABCA10 | 3.925597 | 2.96E-04 | 0.02558 | 5.29 | up |
| GNAZ | 3.916128 | 6.30E-05 | 0.01088 | 6.38 | up |
| MMP23A | 3.915779 | 8.99E-05 | 0.0131 | 6.12 | up |
| CALB2 | 3.877864 | 6.48E-05 | 0.01106 | 6.36 | up |
| NRK | 3.86679 | 1.02E-04 | 0.01412 | 6.03 | up |
| FMO1 | 3.86129 | 1.33E-04 | 0.01622 | 5.84 | up |
| DIRAS3 | 3.861099 | 6.67E-05 | 0.01118 | 6.34 | up |
| BMP3 | 3.860396 | 4.62E-04 | 0.03295 | 4.99 | up |
| KIAA1975 | 3.830097 | 8.26E-05 | 0.01253 | 6.18 | up |
| IGLV3-21 | 3.827557 | 2.85E-04 | 0.02513 | 5.31 | up |
| NTRK2 | 3.797685 | 1.93E-04 | 0.02022 | 5.58 | up |
| SCG2 | 3.793779 | 1.24E-04 | 0.01579 | 5.89 | up |
| SERPINE2 | 3.758574 | 9.28E-05 | 0.01328 | 6.1 | up |
| HOXC4 | 3.750058 | 1.57E-04 | 0.0178 | 5.72 | up |
| HS6ST2 | 3.749942 | 4.04E-04 | 0.03062 | 5.08 | up |
| SPRR2E | 3.749052 | 7.66E-05 | 0.01212 | 6.24 | up |
| MSC | 3.748344 | 1.08E-04 | 0.01488 | 5.99 | up |
| ABCA9 | 3.744803 | 3.29E-04 | 0.02716 | 5.22 | up |
| CDH23 | 3.744571 | 1.06E-04 | 0.01469 | 6 | up |
| FZD7 | 3.734015 | 1.79E-04 | 0.01937 | 5.63 | up |
| PHACTR3 | 3.728861 | 1.37E-04 | 0.01642 | 5.82 | up |
| INHA | 3.717483 | 1.76E-04 | 0.01909 | 5.64 | up |
| LTF | 3.701918 | 1.12E-04 | 0.01516 | 5.97 | up |
| ADH1C | 3.700514 | 9.15E-05 | 0.01321 | 6.11 | up |
| TNNT2 | 3.699832 | 9.72E-05 | 0.01375 | 6.07 | up |
| CILP2 | 3.696656 | 1.10E-04 | 0.01508 | 5.97 | up |
| LOC338328 | 3.687451 | 1.21E-04 | 0.01565 | 5.91 | up |
| TNNI3 | 3.6809 | 1.48E-04 | 0.01728 | 5.77 | up |
| TCERG1L | 3.671632 | 5.86E-04 | 0.03876 | 4.84 | up |
| INMT | 3.657485 | 1.31E-04 | 0.01615 | 5.85 | up |
| CFH | 3.625405 | 1.17E-04 | 0.01528 | 5.93 | up |
| DSCR1L1 | 3.594436 | 4.60E-04 | 0.03292 | 5 | up |
| RYR2 | 3.571354 | 2.04E-04 | 0.02081 | 5.54 | up |
| IL1RAPL1 | 3.552177 | 2.02E-04 | 0.02081 | 5.55 | up |
| DPYSL3 | 3.538412 | 5.40E-04 | 0.03651 | 4.89 | up |
| PCSK2 | 3.518713 | 1.40E-04 | 0.01663 | 5.81 | up |
| ARHGAP22 | 3.50856 | 1.31E-04 | 0.01615 | 5.85 | up |
| SELP | 3.505574 | 1.48E-04 | 0.01728 | 5.77 | up |
| HOXC8 | 3.498043 | 2.93E-04 | 0.02549 | 5.3 | up |
| GSTM3 | 3.491931 | 2.12E-04 | 0.02126 | 5.52 | up |
| JPH2 | 3.490558 | 1.69E-04 | 0.01855 | 5.67 | up |
| NRP2 | 3.482357 | 1.82E-04 | 0.01944 | 5.62 | up |
| IGLV3-25 | 3.448464 | 3.26E-04 | 0.02716 | 5.22 | up |
| GJA5 | 3.445012 | 2.22E-04 | 0.02175 | 5.48 | up |
| GSTA3 | 3.441396 | 1.89E-04 | 0.01989 | 5.6 | up |
| TXLNB | 3.436302 | 7.49E-04 | 0.04488 | 4.68 | up |
| COL11A1 | 3.430367 | 5.92E-04 | 0.0388 | 4.83 | up |
| FOXC1 | 3.411598 | 2.29E-04 | 0.02191 | 5.46 | up |
| FHL5 | 3.411069 | 2.27E-04 | 0.02187 | 5.47 | up |
| LOC145820 | 3.409873 | 7.57E-04 | 0.04488 | 4.68 | up |
| GATM | 3.400478 | 4.39E-04 | 0.03207 | 5.03 | up |
| KLF2 | 3.393819 | 2.44E-04 | 0.02272 | 5.42 | up |
| GIMAP8 | 3.390377 | 2.99E-04 | 0.02578 | 5.28 | up |
| BNC2 | 3.378777 | 2.14E-04 | 0.02128 | 5.51 | up |
| FRZB | 3.357889 | 2.40E-04 | 0.02258 | 5.43 | up |
| C20orf185 | 3.357753 | 4.03E-04 | 0.03062 | 5.08 | up |
| PDE2A | 3.354045 | 2.06E-04 | 0.02095 | 5.53 | up |
| KCNMB1 | 3.35302 | 1.87E-04 | 0.01978 | 5.6 | up |
| LHX9 | 3.348243 | 8.39E-04 | 0.04814 | 4.61 | up |
| AQP1 | 3.338298 | 2.14E-04 | 0.02128 | 5.51 | up |
| PGM5 | 3.329235 | 2.94E-04 | 0.02549 | 5.29 | up |
| CHGB | 3.328555 | 2.14E-04 | 0.02128 | 5.51 | up |
| CCL14 | 3.322914 | 2.69E-04 | 0.02421 | 5.35 | up |
| GAP43 | 3.315311 | 2.87E-04 | 0.02519 | 5.31 | up |
| STAB2 | 3.295694 | 2.22E-04 | 0.02175 | 5.48 | up |
| FRMPD3 | 3.289022 | 3.92E-04 | 0.03028 | 5.1 | up |
| NMNAT3 | 3.274261 | 6.93E-04 | 0.04274 | 4.73 | up |
| ADRB3 | 3.246982 | 3.31E-04 | 0.02716 | 5.21 | up |
| SPRR2B | 3.242063 | 3.62E-04 | 0.02873 | 5.15 | up |
| BDNF | 3.238175 | 3.37E-04 | 0.02749 | 5.2 | up |
| AGC1 | 3.235254 | 4.15E-04 | 0.03115 | 5.06 | up |
| ODZ4 | 3.234269 | 3.66E-04 | 0.02883 | 5.15 | up |
| RGS9 | 3.215553 | 2.65E-04 | 0.02409 | 5.36 | up |
| SMPX | 3.212146 | 4.00E-04 | 0.03062 | 5.09 | up |
| IGHA1 | 3.208743 | 5.88E-04 | 0.03876 | 4.84 | up |
| COL8A1 | 3.188853 | 6.87E-04 | 0.04257 | 4.74 | up |
| PLA2G1B | 3.185189 | 2.76E-04 | 0.02465 | 5.34 | up |
| PODN | 3.180243 | 5.35E-04 | 0.03635 | 4.9 | up |
| KIAA0644 | 3.169297 | 7.86E-04 | 0.04625 | 4.65 | up |
| PON3 | 3.168708 | 6.16E-04 | 0.03951 | 4.81 | up |
| ABCA6 | 3.165421 | 3.51E-04 | 0.02825 | 5.18 | up |
| LOC642891 | 3.155148 | 7.05E-04 | 0.04303 | 4.72 | up |
| AOC3 | 3.154696 | 4.13E-04 | 0.03114 | 5.07 | up |
| MYOZ3 | 3.129213 | 3.40E-04 | 0.0276 | 5.2 | up |
| CASQ2 | 3.111444 | 7.52E-04 | 0.04488 | 4.68 | up |
| TMEM130 | 3.096861 | 4.36E-04 | 0.03197 | 5.03 | up |
| LOC643361 | 3.092156 | 4.92E-04 | 0.03423 | 4.95 | up |
| FST | 3.077471 | 3.76E-04 | 0.02938 | 5.13 | up |
| SORCS2 | 3.063489 | 3.92E-04 | 0.03028 | 5.1 | up |
| AQP11 | 3.06239 | 4.61E-04 | 0.03292 | 5 | up |
| LAG3 | 3.055439 | 4.66E-04 | 0.03297 | 4.99 | up |
| PRSS35 | 3.049617 | 5.58E-04 | 0.03742 | 4.87 | up |
| PLA2G2A | 3.043359 | 8.37E-04 | 0.04812 | 4.61 | up |
| CHRM3 | 3.036469 | 5.07E-04 | 0.03493 | 4.93 | up |
| SLC4A3 | 3.02826 | 5.70E-04 | 0.038 | 4.86 | up |
| WNT2B | 3.025586 | 4.61E-04 | 0.03292 | 5 | up |
| COL8A2 | 3.012661 | 4.41E-04 | 0.0321 | 5.03 | up |
| LOC283174 | 2.991916 | 4.46E-04 | 0.03236 | 5.02 | up |
| HS3ST2 | 2.965791 | 5.38E-04 | 0.0365 | 4.9 | up |
| THBS4 | 2.965001 | 5.24E-04 | 0.03582 | 4.91 | up |
| PTPN5 | 2.957871 | 7.93E-04 | 0.04655 | 4.65 | up |
| GSTM5 | 2.953894 | 5.00E-04 | 0.03471 | 4.94 | up |
| EPHA4 | 2.933434 | 8.46E-04 | 0.04838 | 4.61 | up |
| FHL2 | 2.931193 | 7.20E-04 | 0.04375 | 4.71 | up |
| FCGR2B | 2.920034 | 8.09E-04 | 0.0471 | 4.63 | up |
| CYP4F12 | 2.910633 | 6.99E-04 | 0.04289 | 4.73 | up |
| MMP1 | 2.904901 | 8.79E-04 | 0.04948 | 4.58 | up |
| SCN4B | 2.891162 | 7.34E-04 | 0.04428 | 4.7 | up |
| CLEC4M | 2.891105 | 6.02E-04 | 0.03917 | 4.82 | up |
| PKNOX2 | 2.876379 | 6.07E-04 | 0.03917 | 4.82 | up |
| KRTAP10-1 | 2.869617 | 6.49E-04 | 0.04077 | 4.77 | up |
| LTBP2 | 2.864099 | 8.66E-04 | 0.04903 | 4.59 | up |
| LOC644150 | 2.861228 | 6.79E-04 | 0.04223 | 4.75 | up |
| INHBA | 2.816604 | 8.42E-04 | 0.04824 | 4.61 | up |
| SCARB1 | 2.811676 | 8.68E-04 | 0.04908 | 4.59 | up |
| C10orf54 | 2.799594 | 8.80E-04 | 0.04951 | 4.58 | up |
| BST2 | 2.780287 | 8.05E-04 | 0.04702 | 4.64 | up |
| FXYD1 | 2.769695 | 8.75E-04 | 0.04935 | 4.59 | up |
| CNDP2 | -2.7364 | 8.83E-04 | 0.04962 | -4.58 | down |
| LIMS3 | -2.7441 | 8.52E-04 | 0.04862 | -4.6 | down |
| C21orf121 | -2.76985 | 8.76E-04 | 0.04935 | -4.58 | down |
| CHIA | -2.78222 | 7.98E-04 | 0.04673 | -4.64 | down |
| LOC284422 | -2.8183 | 7.34E-04 | 0.04428 | -4.7 | down |
| MTL5 | -2.82358 | 8.26E-04 | 0.0477 | -4.62 | down |
| EPHA1 | -2.82799 | 8.89E-04 | 0.04982 | -4.58 | down |
| SLC7A1 | -2.83776 | 7.72E-04 | 0.04565 | -4.66 | down |
| TMPRSS4 | -2.85171 | 7.30E-04 | 0.04423 | -4.7 | down |
| ATP2B2 | -2.86144 | 8.30E-04 | 0.04784 | -4.62 | down |
| SLC16A6 | -2.87352 | 7.56E-04 | 0.04488 | -4.68 | down |
| SHANK2 | -2.88159 | 8.43E-04 | 0.04824 | -4.61 | down |
| SOX17 | -2.88185 | 6.48E-04 | 0.04077 | -4.78 | down |
| FAM83H | -2.88776 | 6.21E-04 | 0.03971 | -4.8 | down |
| PGBD5 | -2.89217 | 5.94E-04 | 0.0388 | -4.83 | down |
| TLCD1 | -2.92144 | 6.33E-04 | 0.04024 | -4.79 | down |
| LOC642934 | -2.92775 | 5.88E-04 | 0.03876 | -4.84 | down |
| AQP5 | -2.93394 | 7.53E-04 | 0.04488 | -4.68 | down |
| TSGA2 | -2.97213 | 5.55E-04 | 0.0373 | -4.88 | down |
| SERPINA6 | -2.9792 | 4.88E-04 | 0.03411 | -4.96 | down |
| RBM35B | -2.99131 | 5.22E-04 | 0.03578 | -4.91 | down |
| DSCR1L2 | -3.00036 | 4.39E-04 | 0.03207 | -5.03 | down |
| ACSL5 | -3.00769 | 5.28E-04 | 0.03594 | -4.91 | down |
| SLC18A2 | -3.00825 | 6.00E-04 | 0.03911 | -4.82 | down |
| INDOL1 | -3.01083 | 6.08E-04 | 0.03917 | -4.82 | down |
| ECM1 | -3.0124 | 4.79E-04 | 0.03354 | -4.97 | down |
| WWC1 | -3.03666 | 5.61E-04 | 0.03758 | -4.87 | down |
| ST14 | -3.04645 | 5.39E-04 | 0.0365 | -4.89 | down |
| C9orf71 | -3.07156 | 4.68E-04 | 0.03305 | -4.99 | down |
| SLC28A2 | -3.0722 | 6.63E-04 | 0.04153 | -4.76 | down |
| CEACAM1 | -3.07633 | 4.72E-04 | 0.03323 | -4.98 | down |
| SMPDL3B | -3.07944 | 3.77E-04 | 0.0294 | -5.13 | down |
| SFN | -3.08906 | 7.64E-04 | 0.04518 | -4.67 | down |
| ADAMTS6 | -3.09685 | 8.06E-04 | 0.04702 | -4.64 | down |
| PLLP | -3.10096 | 3.55E-04 | 0.02833 | -5.17 | down |
| CRLF1 | -3.10236 | 6.14E-04 | 0.03949 | -4.81 | down |
| DNAH5 | -3.10355 | 4.17E-04 | 0.03124 | -5.06 | down |
| GPR110 | -3.10515 | 7.86E-04 | 0.04625 | -4.65 | down |
| LOC222171 | -3.12682 | 8.31E-04 | 0.04789 | -4.62 | down |
| GATA2 | -3.12834 | 6.53E-04 | 0.0409 | -4.77 | down |
| FLJ30046 | -3.13128 | 6.28E-04 | 0.04003 | -4.8 | down |
| HAP1 | -3.14124 | 8.89E-04 | 0.04982 | -4.58 | down |
| PTGS1 | -3.14612 | 3.16E-04 | 0.02677 | -5.24 | down |
| B4GALNT2 | -3.16019 | 3.98E-04 | 0.03053 | -5.09 | down |
| ST6GALNAC1 | -3.16319 | 3.23E-04 | 0.02716 | -5.23 | down |
| MAP2K6 | -3.16567 | 4.66E-04 | 0.03297 | -4.99 | down |
| C4orf19 | -3.16578 | 3.08E-04 | 0.02634 | -5.26 | down |
| MCOLN3 | -3.16829 | 6.66E-04 | 0.04164 | -4.76 | down |
| FLJ46082 | -3.17366 | 3.39E-04 | 0.0276 | -5.2 | down |
| DSU | -3.17678 | 7.55E-04 | 0.04488 | -4.68 | down |
| WDR16 | -3.17781 | 3.31E-04 | 0.02716 | -5.21 | down |
| C1orf172 | -3.17874 | 2.94E-04 | 0.02549 | -5.29 | down |
| LRRC1 | -3.17893 | 6.52E-04 | 0.0409 | -4.77 | down |
| HPN | -3.18117 | 4.30E-04 | 0.03169 | -5.04 | down |
| DEFB124 | -3.19698 | 2.81E-04 | 0.02482 | -5.32 | down |
| RAB11FIP4 | -3.19952 | 3.28E-04 | 0.02716 | -5.22 | down |
| FAM81B | -3.1996 | 2.67E-04 | 0.02415 | -5.36 | down |
| MT1G | -3.20715 | 2.61E-04 | 0.02389 | -5.37 | down |
| ROPN1L | -3.21953 | 3.09E-04 | 0.02634 | -5.26 | down |
| GCNT1 | -3.21968 | 5.11E-04 | 0.0352 | -4.93 | down |
| TMEM101 | -3.22701 | 4.33E-04 | 0.0318 | -5.04 | down |
| LOC553158 | -3.23263 | 2.71E-04 | 0.02427 | -5.35 | down |
| CDYL2 | -3.23617 | 3.11E-04 | 0.02649 | -5.26 | down |
| GPR160 | -3.23787 | 6.73E-04 | 0.04196 | -4.75 | down |
| HOXB9 | -3.23848 | 2.72E-04 | 0.02427 | -5.35 | down |
| RBM24 | -3.24443 | 3.32E-04 | 0.02716 | -5.21 | down |
| OLIG3 | -3.24813 | 2.88E-04 | 0.02525 | -5.31 | down |
| GRHL2 | -3.25214 | 3.44E-04 | 0.02775 | -5.19 | down |
| KCNK13 | -3.25317 | 3.19E-04 | 0.02696 | -5.24 | down |
| TPD52L1 | -3.25343 | 3.41E-04 | 0.0276 | -5.2 | down |
| C1orf186 | -3.26288 | 2.27E-04 | 0.02187 | -5.47 | down |
| PRRG4 | -3.27359 | 4.22E-04 | 0.03149 | -5.05 | down |
| RNF183 | -3.27638 | 4.46E-04 | 0.03236 | -5.02 | down |
| CYP2J2 | -3.28155 | 3.76E-04 | 0.02938 | -5.13 | down |
| ITIH2 | -3.2841 | 3.14E-04 | 0.02666 | -5.25 | down |
| LOC646267 | -3.28953 | 2.45E-04 | 0.02272 | -5.42 | down |
| MFSD2 | -3.29484 | 2.10E-04 | 0.02117 | -5.52 | down |
| IRF6 | -3.29665 | 2.56E-04 | 0.02353 | -5.39 | down |
| DNAH11 | -3.3024 | 2.78E-04 | 0.02473 | -5.33 | down |
| LAD1 | -3.30586 | 3.20E-04 | 0.027 | -5.24 | down |
| SLC34A2 | -3.30744 | 3.16E-04 | 0.02677 | -5.25 | down |
| MGC42090 | -3.30847 | 4.65E-04 | 0.03297 | -4.99 | down |
| ATP6V1C2 | -3.31378 | 6.04E-04 | 0.03917 | -4.82 | down |
| ARHGAP8 | -3.31381 | 2.08E-04 | 0.02111 | -5.53 | down |
| SLC23A1 | -3.31845 | 2.28E-04 | 0.0219 | -5.47 | down |
| RP13-347D8.3 | -3.32645 | 4.96E-04 | 0.0345 | -4.95 | down |
| GLYATL2 | -3.33012 | 4.79E-04 | 0.03354 | -4.97 | down |
| C1orf210 | -3.33095 | 2.54E-04 | 0.02342 | -5.39 | down |
| TRPV6 | -3.34062 | 2.00E-04 | 0.0207 | -5.55 | down |
| COBL | -3.34154 | 3.31E-04 | 0.02716 | -5.21 | down |
| DCDC2 | -3.34881 | 4.72E-04 | 0.03323 | -4.98 | down |
| LOC644980 | -3.35583 | 3.55E-04 | 0.02833 | -5.17 | down |
| SLCO4A1 | -3.36237 | 1.80E-04 | 0.01937 | -5.63 | down |
| C1orf64 | -3.36494 | 1.75E-04 | 0.01907 | -5.65 | down |
| KCTD14 | -3.36605 | 8.51E-04 | 0.0486 | -4.6 | down |
| TACSTD1 | -3.37125 | 3.94E-04 | 0.03033 | -5.1 | down |
| FOXJ1 | -3.37955 | 4.03E-04 | 0.03062 | -5.08 | down |
| DUOX1 | -3.37962 | 5.24E-04 | 0.03582 | -4.91 | down |
| PLCB1 | -3.38002 | 6.21E-04 | 0.03971 | -4.8 | down |
| NLF2 | -3.38385 | 4.50E-04 | 0.03251 | -5.01 | down |
| FLJ40919 | -3.39545 | 2.41E-04 | 0.02258 | -5.43 | down |
| KIAA1456 | -3.39694 | 4.24E-04 | 0.0315 | -5.05 | down |
| CYP4B1 | -3.39742 | 1.66E-04 | 0.01842 | -5.68 | down |
| PLS1 | -3.40108 | 4.10E-04 | 0.03097 | -5.07 | down |
| UBXD3 | -3.40433 | 2.30E-04 | 0.02191 | -5.46 | down |
| ENPP4 | -3.4135 | 5.55E-04 | 0.0373 | -4.88 | down |
| C1orf34 | -3.41588 | 1.82E-04 | 0.01944 | -5.62 | down |
| TRPM6 | -3.42169 | 4.47E-04 | 0.03238 | -5.02 | down |
| CHMP4C | -3.42561 | 1.95E-04 | 0.02028 | -5.57 | down |
| DSPG3 | -3.43254 | 6.05E-04 | 0.03917 | -4.82 | down |
| LOC440895 | -3.44337 | 2.47E-04 | 0.02288 | -5.41 | down |
| SST | -3.44929 | 4.02E-04 | 0.03062 | -5.09 | down |
| VTCN1 | -3.4642 | 3.54E-04 | 0.02833 | -5.17 | down |
| EDG7 | -3.46976 | 2.09E-04 | 0.02111 | -5.53 | down |
| SCNN1G | -3.47359 | 1.52E-04 | 0.01742 | -5.75 | down |
| EDAR | -3.4736 | 2.03E-04 | 0.02081 | -5.55 | down |
| KLK4 | -3.47591 | 2.45E-04 | 0.02272 | -5.42 | down |
| MOXD1 | -3.48486 | 5.75E-04 | 0.03817 | -4.85 | down |
| TEKT1 | -3.48691 | 2.09E-04 | 0.02111 | -5.52 | down |
| TMC4 | -3.49225 | 3.57E-04 | 0.02844 | -5.16 | down |
| EVA1 | -3.5004 | 3.40E-04 | 0.0276 | -5.2 | down |
| CLDN7 | -3.50847 | 1.66E-04 | 0.01842 | -5.69 | down |
| C9orf24 | -3.52155 | 2.25E-04 | 0.02187 | -5.47 | down |
| SLC5A9 | -3.52355 | 1.76E-04 | 0.01907 | -5.65 | down |
| ACPP | -3.52438 | 1.52E-04 | 0.01742 | -5.75 | down |
| LOC138255 | -3.55662 | 5.04E-04 | 0.03486 | -4.94 | down |
| IL20RA | -3.55889 | 4.89E-04 | 0.03412 | -4.96 | down |
| MUC15 | -3.56365 | 2.68E-04 | 0.02415 | -5.36 | down |
| PERP | -3.57149 | 1.28E-04 | 0.01593 | -5.87 | down |
| TMEM132B | -3.57311 | 7.15E-04 | 0.04349 | -4.71 | down |
| CDH1 | -3.58919 | 1.26E-04 | 0.01587 | -5.88 | down |
| GDA | -3.61463 | 7.55E-04 | 0.04488 | -4.68 | down |
| SPAG6 | -3.61635 | 4.34E-04 | 0.03189 | -5.03 | down |
| CDCP1 | -3.62556 | 9.56E-05 | 0.01359 | -6.08 | down |
| RORC | -3.62694 | 1.13E-04 | 0.01516 | -5.95 | down |
| PTPN3 | -3.63264 | 3.07E-04 | 0.02634 | -5.27 | down |
| BSPRY | -3.63318 | 1.30E-04 | 0.01608 | -5.86 | down |
| PCK1 | -3.63616 | 9.80E-05 | 0.01378 | -6.06 | down |
| CLDN4 | -3.63651 | 2.66E-04 | 0.02415 | -5.36 | down |
| TMEM28 | -3.65771 | 1.67E-04 | 0.01844 | -5.68 | down |
| MYB | -3.66067 | 1.59E-04 | 0.01796 | -5.71 | down |
| BTBD11 | -3.67968 | 1.28E-04 | 0.01592 | -5.87 | down |
| STXBP2 | -3.68053 | 2.04E-04 | 0.02081 | -5.54 | down |
| RIPK4 | -3.68054 | 3.69E-04 | 0.02899 | -5.14 | down |
| LRRC8E | -3.69209 | 1.40E-04 | 0.01663 | -5.8 | down |
| C1orf158 | -3.69898 | 1.22E-04 | 0.01565 | -5.9 | down |
| HOXA5 | -3.7086 | 2.27E-04 | 0.02187 | -5.47 | down |
| NRXN3 | -3.71814 | 1.40E-04 | 0.01663 | -5.8 | down |
| C1orf173 | -3.71836 | 3.07E-04 | 0.02634 | -5.26 | down |
| MGC16372 | -3.71886 | 1.14E-04 | 0.01516 | -5.95 | down |
| CA8 | -3.73078 | 7.41E-04 | 0.04463 | -4.69 | down |
| CYP26A1 | -3.78212 | 6.70E-05 | 0.01118 | -6.34 | down |
| XDH | -3.78475 | 7.09E-04 | 0.04319 | -4.72 | down |
| PLCH1 | -3.7857 | 5.95E-04 | 0.0388 | -4.83 | down |
| ACCN1 | -3.79253 | 6.58E-05 | 0.01114 | -6.35 | down |
| PKD1L2 | -3.79577 | 6.71E-05 | 0.01118 | -6.34 | down |
| OPRK1 | -3.79765 | 1.15E-04 | 0.01516 | -5.95 | down |
| CST1 | -3.79954 | 8.23E-05 | 0.01252 | -6.19 | down |
| STXBP6 | -3.80026 | 9.79E-05 | 0.01378 | -6.06 | down |
| GCNT3 | -3.80116 | 1.34E-04 | 0.01622 | -5.84 | down |
| BCAS1 | -3.83104 | 7.55E-05 | 0.01207 | -6.25 | down |
| DEFB1 | -3.84736 | 1.15E-04 | 0.01516 | -5.94 | down |
| OSTbeta | -3.84981 | 7.59E-05 | 0.01209 | -6.25 | down |
| HOXB5 | -3.8542 | 5.82E-05 | 0.01038 | -6.44 | down |
| EXPH5 | -3.8593 | 1.50E-04 | 0.01735 | -5.76 | down |
| MPPED2 | -3.86183 | 3.44E-04 | 0.02775 | -5.19 | down |
| GALNT12 | -3.87023 | 8.10E-04 | 0.0471 | -4.63 | down |
| CXADR | -3.87131 | 1.28E-04 | 0.01592 | -5.87 | down |
| LOC644612 | -3.87539 | 7.78E-05 | 0.01223 | -6.23 | down |
| UGT2B7 | -3.88196 | 1.43E-04 | 0.01686 | -5.79 | down |
| ATAD4 | -3.89357 | 6.08E-05 | 0.01064 | -6.41 | down |
| DLGAP1 | -3.89905 | 1.12E-04 | 0.01516 | -5.96 | down |
| RAB25 | -3.89939 | 5.25E-04 | 0.03584 | -4.91 | down |
| RHPN2 | -3.90034 | 1.54E-04 | 0.01759 | -5.74 | down |
| FGFR2 | -3.9024 | 1.13E-04 | 0.01516 | -5.95 | down |
| TMEM16A | -3.90475 | 5.27E-05 | 0.00999 | -6.52 | down |
| FLJ44379 | -3.9146 | 2.27E-04 | 0.02187 | -5.47 | down |
| LOC642299 | -3.91711 | 6.11E-05 | 0.01066 | -6.41 | down |
| PHYHIPL | -3.92625 | 6.91E-04 | 0.04272 | -4.74 | down |
| HOXB3 | -3.92698 | 8.38E-05 | 0.01267 | -6.17 | down |
| SERPINA4 | -3.96108 | 5.44E-05 | 0.01004 | -6.5 | down |
| DNHD2 | -3.97705 | 2.93E-04 | 0.02549 | -5.3 | down |
| HOXB8 | -3.98853 | 4.84E-05 | 0.0098 | -6.58 | down |
| AGR2 | -4.00445 | 2.15E-04 | 0.02128 | -5.51 | down |
| PPAP2C | -4.02049 | 6.01E-05 | 0.01059 | -6.42 | down |
| RASEF | -4.02098 | 7.91E-05 | 0.01236 | -6.22 | down |
| MARVELD2 | -4.02448 | 1.27E-04 | 0.01592 | -5.87 | down |
| ARMC3 | -4.02478 | 2.15E-04 | 0.02128 | -5.51 | down |
| SCNN1A | -4.02844 | 1.17E-04 | 0.01528 | -5.93 | down |
| NPAS3 | -4.02894 | 4.29E-05 | 0.00934 | -6.68 | down |
| LRRC31 | -4.02982 | 9.08E-05 | 0.01314 | -6.12 | down |
| SLITRK6 | -4.0444 | 1.51E-04 | 0.01742 | -5.75 | down |
| C12orf27 | -4.05061 | 6.26E-05 | 0.01085 | -6.39 | down |
| PGR | -4.05174 | 5.05E-04 | 0.03487 | -4.94 | down |
| EDN3 | -4.06007 | 9.63E-05 | 0.01365 | -6.07 | down |
| CAPSL | -4.06446 | 1.01E-04 | 0.01404 | -6.04 | down |
| LOC646201 | -4.08436 | 8.65E-05 | 0.01287 | -6.15 | down |
| HOXB4 | -4.10131 | 7.20E-05 | 0.01167 | -6.29 | down |
| GREM2 | -4.11762 | 9.02E-05 | 0.0131 | -6.12 | down |
| LOC644715 | -4.11898 | 5.15E-05 | 0.00998 | -6.54 | down |
| ESR1 | -4.13318 | 5.39E-05 | 0.01004 | -6.5 | down |
| HAL | -4.14584 | 4.79E-05 | 0.00979 | -6.59 | down |
| GAL | -4.15116 | 3.20E-05 | 0.00777 | -6.9 | down |
| ERBB3 | -4.16056 | 3.32E-05 | 0.0079 | -6.88 | down |
| IRX3 | -4.16236 | 4.40E-05 | 0.00942 | -6.66 | down |
| MFAP3L | -4.16751 | 5.46E-05 | 0.01004 | -6.49 | down |
| MUC13 | -4.16982 | 1.00E-04 | 0.01401 | -6.04 | down |
| IHH | -4.1838 | 1.22E-04 | 0.01565 | -5.9 | down |
| CA12 | -4.19155 | 2.72E-05 | 0.00738 | -7.03 | down |
| TCN1 | -4.20068 | 1.21E-04 | 0.01565 | -5.91 | down |
| WFDC2 | -4.21037 | 1.66E-04 | 0.01842 | -5.69 | down |
| BCMP11 | -4.21675 | 3.58E-04 | 0.02846 | -5.16 | down |
| SERINC2 | -4.2184 | 1.41E-04 | 0.01667 | -5.8 | down |
| ILDR1 | -4.22175 | 5.63E-05 | 0.01008 | -6.47 | down |
| TMPRSS2 | -4.23611 | 3.19E-05 | 0.00777 | -6.91 | down |
| SALL1 | -4.23666 | 2.59E-05 | 0.00724 | -7.07 | down |
| HOXB7 | -4.23991 | 2.94E-05 | 0.0074 | -6.97 | down |
| MME | -4.24613 | 3.11E-05 | 0.00774 | -6.93 | down |
| FXYD3 | -4.30216 | 5.63E-05 | 0.01008 | -6.47 | down |
| HOOK1 | -4.33213 | 7.46E-05 | 0.012 | -6.26 | down |
| NIP | -4.33361 | 2.82E-05 | 0.00738 | -7 | down |
| FGL1 | -4.3387 | 4.70E-05 | 0.00969 | -6.61 | down |
| FLJ10847 | -4.34273 | 2.25E-05 | 0.00684 | -7.19 | down |
| B4GALNT3 | -4.34921 | 2.25E-05 | 0.00684 | -7.18 | down |
| HOMER2 | -4.36025 | 2.85E-05 | 0.00738 | -7 | down |
| OVOL2 | -4.36847 | 2.78E-05 | 0.00738 | -7.01 | down |
| GP2 | -4.37173 | 2.16E-05 | 0.00676 | -7.22 | down |
| UPK1B | -4.37787 | 6.91E-05 | 0.01139 | -6.32 | down |
| SLC15A1 | -4.3827 | 3.09E-05 | 0.00774 | -6.93 | down |
| HOXB6 | -4.39381 | 2.43E-05 | 0.00707 | -7.12 | down |
| TCF2 | -4.40003 | 1.81E-04 | 0.01937 | -5.63 | down |
| MET | -4.41704 | 1.10E-04 | 0.01508 | -5.97 | down |
| SLC15A2 | -4.44417 | 4.50E-05 | 0.00952 | -6.64 | down |
| LOC283514 | -4.4608 | 5.07E-05 | 0.00998 | -6.55 | down |
| GLT1D1 | -4.47502 | 2.84E-05 | 0.00738 | -7 | down |
| ESPN | -4.47924 | 1.53E-05 | 0.00536 | -7.5 | down |
| ASRGL1 | -4.48397 | 3.39E-05 | 0.00802 | -6.86 | down |
| LGR7 | -4.49567 | 2.34E-04 | 0.02212 | -5.45 | down |
| EYA2 | -4.5117 | 1.53E-05 | 0.00536 | -7.5 | down |
| FLJ37927 | -4.51317 | 2.30E-04 | 0.02191 | -5.46 | down |
| RP5-1065J22.5 | -4.51727 | 1.08E-04 | 0.01488 | -5.99 | down |
| GJB6 | -4.53385 | 1.15E-04 | 0.01516 | -5.95 | down |
| PAEP | -4.5572 | 1.34E-05 | 0.00531 | -7.61 | down |
| PAX2 | -4.56303 | 1.96E-05 | 0.00639 | -7.3 | down |
| HPSE2 | -4.57116 | 2.22E-05 | 0.00684 | -7.19 | down |
| ATP8B3 | -4.57662 | 1.61E-05 | 0.00553 | -7.46 | down |
| DMBT1 | -4.58249 | 1.49E-05 | 0.00536 | -7.52 | down |
| HOXA9 | -4.58294 | 1.62E-04 | 0.01816 | -5.7 | down |
| HOXA10 | -4.61894 | 1.22E-05 | 0.005 | -7.69 | down |
| AP1M2 | -4.64237 | 1.43E-05 | 0.00536 | -7.56 | down |
| DLX5 | -4.6429 | 1.31E-05 | 0.00523 | -7.63 | down |
| DIO2 | -4.66152 | 3.41E-05 | 0.00803 | -6.85 | down |
| KIAA0703 | -4.79188 | 1.69E-05 | 0.00566 | -7.42 | down |
| KCNC3 | -4.82264 | 9.06E-06 | 0.00411 | -7.95 | down |
| CXCL14 | -4.83108 | 1.45E-05 | 0.00536 | -7.55 | down |
| GRAMD1C | -4.84937 | 2.82E-04 | 0.0249 | -5.32 | down |
| SLC30A2 | -4.8887 | 1.40E-05 | 0.00536 | -7.57 | down |
| ORM2 | -4.89207 | 6.96E-06 | 0.00375 | -8.18 | down |
| CXCL13 | -4.89979 | 6.61E-05 | 0.01114 | -6.35 | down |
| LOC642419 | -4.91275 | 8.39E-06 | 0.00403 | -8.01 | down |
| CRISP3 | -4.91764 | 5.45E-05 | 0.01004 | -6.49 | down |
| CDCA7 | -4.94381 | 3.80E-04 | 0.0295 | -5.12 | down |
| MAL2 | -4.9626 | 1.95E-05 | 0.00639 | -7.3 | down |
| GJB1 | -4.99362 | 6.64E-06 | 0.00375 | -8.22 | down |
| CPM | -4.99647 | 1.46E-05 | 0.00536 | -7.54 | down |
| DLX6 | -4.99649 | 6.73E-06 | 0.00375 | -8.21 | down |
| SLC3A1 | -5.01009 | 1.04E-05 | 0.00446 | -7.83 | down |
| PKHD1L1 | -5.05472 | 1.31E-05 | 0.00523 | -7.63 | down |
| C20orf85 | -5.05506 | 6.81E-06 | 0.00375 | -8.2 | down |
| GALNT4 | -5.07962 | 3.90E-05 | 0.00885 | -6.75 | down |
| MSX1 | -5.08149 | 4.93E-06 | 0.00345 | -8.49 | down |
| FLJ21511 | -5.09886 | 7.33E-06 | 0.0038 | -8.13 | down |
| SPDEF | -5.12126 | 2.91E-05 | 0.0074 | -6.98 | down |
| HSD17B2 | -5.12758 | 9.67E-06 | 0.00428 | -7.89 | down |
| TSCOT | -5.14567 | 5.89E-06 | 0.00366 | -8.33 | down |
| RBM35A | -5.15066 | 8.69E-06 | 0.00403 | -7.98 | down |
| PRSS8 | -5.2063 | 6.40E-06 | 0.00375 | -8.25 | down |
| PPP2R2C | -5.28835 | 2.49E-05 | 0.00719 | -7.1 | down |
| MGC13057 | -5.29629 | 3.85E-05 | 0.00881 | -6.76 | down |
| GABRP | -5.32421 | 7.60E-06 | 0.00381 | -8.1 | down |
| C9orf152 | -5.33379 | 5.70E-06 | 0.00366 | -8.36 | down |
| HOXA11 | -5.42443 | 5.64E-06 | 0.00366 | -8.37 | down |
| UGT8 | -5.43647 | 1.60E-05 | 0.00551 | -7.47 | down |
| EHF | -5.45155 | 7.17E-05 | 0.01167 | -6.29 | down |
| LOC205251 | -5.58263 | 2.40E-06 | 0.00248 | -9.17 | down |
| KMO | -5.5928 | 1.43E-05 | 0.00536 | -7.56 | down |
| ALDH3B2 | -5.63818 | 1.79E-06 | 0.0023 | -9.45 | down |
| HGD | -5.73117 | 2.33E-06 | 0.00247 | -9.19 | down |
| TMED6 | -5.80868 | 6.86E-06 | 0.00375 | -8.19 | down |
| ENPP3 | -5.85935 | 6.03E-06 | 0.00366 | -8.31 | down |
| FOXA2 | -5.87268 | 1.25E-06 | 0.00198 | -9.82 | down |
| TRH | -5.94193 | 1.14E-06 | 0.00198 | -9.91 | down |
| MSX2 | -5.98869 | 1.24E-06 | 0.00198 | -9.83 | down |
| OLFM4 | -6.00327 | 3.84E-06 | 0.00305 | -8.72 | down |
| CTNNA2 | -6.03862 | 1.74E-06 | 0.0023 | -9.48 | down |
| ORM1 | -6.34752 | 6.96E-07 | 0.00158 | -1.04E+01 | down |
| MS4A8B | -6.48352 | 4.99E-07 | 0.00125 | -1.08E+01 | down |
| SCGB2A1 | -6.70168 | 2.28E-06 | 0.00247 | -9.22 | down |
| KIAA1324 | -6.94375 | 2.58E-07 | 0.00124 | -1.15E+01 | down |
| SCGB1D4 | -6.99418 | 7.33E-07 | 0.00159 | -1.04E+01 | down |
| SCGB1D2 | -7.17088 | 2.36E-07 | 0.00124 | -1.16E+01 | down |
| MMP26 | -8.03479 | 8.44E-08 | 0.00124 | -1.29E+01 | down |
